# Standard Surgical Approaches to Primary Choledocholithiasis — Definitive Versus Temporary Decompression

**DOI:** 10.1155/1992/38293

**Published:** 1992

**Authors:** António Castro Mendes de Almeida, Fernando José Aldeia, Noel Medina dos Santos, Caetano Winston Gracias

**Affiliations:** Unioersity Hospital of Santa Maria Lisbon Portugal

## Abstract

The occurrence of retained/recurrent calculi after primary CBDE followed by temporary T-tube decompression, have remained at rates varying from 5.4% to 20.9% over the last 10 years in spite of sophisticated pre and intraoperative imaging techniques. It is postulated that a functional obstruction, due to dysmotility of the SO, lies behind most stone-containing ducts. Thus it seems logical to us that a permanent “fenestration” should be the management of most such ducts.

We prospectively followed-up, for one to 10 years, two groups of patients submitted to primary CBDE aiming to assess the short and long-term results of two different surgical approaches to duct lithiasis. In one (Group A) 162 CBDE's were performed, out of 680 CHE's (24%), with a “positivity” of 68% and in the other (Group B) 80 CBDE's, out of 438 CHE's (18%), with a “positivity” of 70%. In Group A a T-tube decompression was used in 79(49%) and a definitive drainage in 83(51%) whereas in Group B the T-tube was employed in only 3(4%) and some form of permanent “fenestration” in 77(96%). There were no significant differences between the operative mortality rates, which were 2.5% in Group A (1 death post T-tube, 3 post CDJ) and 1.3% in Group B (1 death post CDD). The long-term results, though, were significantly worse among patients of Group A whose ducts were temporarily decompressed: 10/79 (12.7%) required further aggressive interventional therapy for retained/recurrent stones while only 3.8% (3/80) in Group A and 1.3% (1/76) in Group B required revisional surgery for bilio-digestive anastomotic complications with cholangitis.

It is concluded that it is against the long-term efficiency of the approach utilized in Group B that the new laparoscopic techniques should be compared.


*HPB Surgery*, 1992, Vol. 6, pp. 35–49
Reprints available directly from the publisher
Photocopying permitted by license only

© 1992 Harwood Academic Publishers GmbH
Printed in the United Kingdom

### STANDARD SURGICAL APPROACHES TO PRIMARY CHOLEDOCHOLITHIASIS — DEFINITIVE VERSUS TEMPORARY DECOMPRESSION*

ANTÓNIO CASTRO MENDES de ALMEIDA, FERNANDO JOSÉ ALDEIA, NOEL MEDINA DOS SANTOS and CAETANO WINSTON GRACIAS

*University Hospital of Santa Maria, Lisbon, Portugal*

*(Received 12 May 1991)*

The occurrence of retained/recurrent calculi after primary CBDE followed by temporary T-tube decompression, have remained at rates varying from 5.4% to 20.9% over the last 10 years in spite of sophisticated pre and intraoperative imaging techniques. It is postulated that a functional obstruction, due to dysmotility of the SO, lies behind most stone-containing ducts. Thus it seems logical to us that a permanent "fenestration" should be the management of most such ducts.

We prospectively followed-up, for one to 10 years, two groups of patients submitted to primary CBDE aiming to assess the short and long-term results of two different surgical approaches to duct lithiasis. In one (Group A) 162 CBDE's were performed, out of 680 CHE's (24%), with a "positivity" of 68% and in the other (Group B) 80 CBDE's, out of 438 CHE's (18%), with a "positivity" of 70%. In Group A a T-tube decompression was used in 79(49%) and a definitive drainage in 83(51%) whereas in Group B the T-tube was employed in only 3(4%) and some form of permanent "fenestration" in 77(96%). There were no significant differences between the operative mortality rates, which were 2.5% in Group A (1 death post T-tube, 3 post CDJ) and 1.3% in Group B (1 death post CDD). The long-term results, though, were significantly worse among patients of Group A whose ducts were temporarily decompressed: 10/79 (12.7%) required further aggressive interventional therapy for retained/recurrent stones while only 3.8% (3/80) in Group A and 1.3% (1/76) in Group B required revisional surgery for bilio-digestive anastomotic complications with cholangitis.

It is concluded that it is against the long-term efficiency of the approach utilized in Group B that the new laparoscopic techniques should be compared.

KEY WORDS: Choledocholithiasis, T-tube drainage, definitive drainage procedures, choledochoduodenostomy

### INTRODUCTION

In 1974, Classen[1] and Kawai[2] first described endoscopic sphincterotomy (EST), with or without intracholedochal mechanical or extra-corporeal shock wave lithotripsy (ESWL). This became widely accepted as the treatment of choice for overlooked, retained or recurrent common bile duct (CBD) stones, irrespective of

Address correspondence to: António Castro Mendes de Almeida, MD, FACS, Rua das Praças, 43 3º Esq., 1200 Lisbon, Portugal.

*This paper was accepted and presented to the World Congress of the WAHPBS, June 1990, London, UK.

the grade of surgical risk. Similarly there is little dispute that a poor risk patient, with *primary* choledocholithiasis, is best managed by EST[3,4]. The issue of subsequently leaving the diseased gallbladder (GB) *in situ* or of removing it remains undecided[3–7].

A different question, still unanswered, is deciding which is the optimal approach for a fit, young, patient requiring *primary* management of CBD stones and/or papillary stenosis. The excessive enthusiasm surrounding the success of EST has led to the erroneous philosophy that anything is good provided it avoids an operation. Indeed, there are studies concluding that "fit patients should be treated by surgery alone without routine preoperative EST"[3], that "endoscopic therapy of CBD calculi does not offer significant advantages with respect to morbidity, mortality and success"[8,9] and that, "an operation may well be the simplest, quickest and even the safest way of removing duct stones"[10].

While the exact roles of the available therapeutic alternatives (EST, ESWL, formal or laparoscopic surgery and dissolution therapy) awaits a definition, resulting from large-scale, long-term, randomized follow-up studies, one issue remains uncontroversial: if surgery is to maintain its status as the dominant method of *primary* treatment of good risk patients with CBD stones it must achieve the lowest possible short and long-term morbidity and mortality rates while leading to the highest possible proportion of long-term symptom-free patients. The optimal performance of surgery is, obviously, of great medical and economic benefit.

Attention to the natural history of CBD stones and the functional status of the "Sphincter of Oddi" is a requirement just as important as suitable technical expertise. In this study, we prospectively followed-up patients submitted to primary common bile duct exploration (CBDE) within the same surgical unit. We specifically aimed to compare two different approaches, based on correspondingly different concepts of the ill understood natural history of choledochal calculi: THEORY I — Assumes that most CBD stones form primarily within the GB, eventually migrating into the CBD[10,11]. If this assumption were true then temporary drainage of the explored ducts, with a T-tube, would be sufficient provided complete duct clearance could be guaranteed. THEORY II — assumes that the majority of duct stones originate "de novo" within the duct itself[12,13] or, having migrated from the GB, remain in the duct growing in size and causing increased pressure to dilate the CBD and the cystic duct, thus facilitating further stone migration[14]. Dysmotility and consequent functional obstruction of the Sphincter of Oddi (SO), hindering the passage of a calculus that was able to negotiate the cystic duct, offers an appealing explanation[15,16]. Were this contention true a permanent "fenestration" of most ducts requiring exploration would be the most sensible approach, because of the risk of retained/recurrent stones.

In so doing we aimed at answering the following questions:

(1) Is there a difference in the percentage of CBDE's and corresponding "positivity" or "negativity" from one approach to the other and in short and long-term morbidity and mortality rates?
(2) Which approach entails less chances of residual/retained/recurrent duct stones and/or need for further invasive treatment?
(3) Which approach leads on to higher rates of long-term symptom-free patients?

### PATIENTS, METHODS

This study relates to the *primary* treatment of gallstones. From January 1980 through December 1989 a total of 1118 cholecystectomies (CHE) were performed within one of the surgical units (four teams, each one led by a consultant surgeon) of a major teaching hospital. Emergency surgery for acute cholecystitis or acute cholangitis was not included nor were CHE's performed as "incidental" procedures in the course of laparotomies meant for pathology other than biliary lithiasis.

A total of 680 CHE's (Group A) were performed by three of the teams according to THEORY I, as shown in Figure 1. The remainder 438 (Group B) were performed by the Senior Author's (ACMA) team, taking THEORY II as correct, as shown in Figure 2 and following previously published guidelines[15,16]. The comparability of patients undergoing CBDE in both Groups, in terms of age and sex distribution and ASA (American Society of Anesthesiology) risk classification is shown in Figures 3 and 4. The preoperative evaluation was similar in both Groups. In Group B, though, particular attention was paid to features pointing to a sub-set of patients whose clinical syndrome or liver function tests (LFT), intravenous cholangiography (IVC) and/or ultrasonographic (USG) findings raised the suspicion of SO dysfunction (Table 1) with the consequent increased likelihood of retention of migrated GB calculi or of stasis and *de novo* formation of stones. As already stressed by us[16], IVC, by demonstrating sluggish duct-duodenal emptying (not by revealing the presence of stones), was particularly helpful in this context. The criteria dictating the need for CBDE, shown in Table 2, were valid in both Groups, although different emphasis was placed on some of the issues from one Group to the other. The major differences rested in the intraoperative evaluation and post-choledocholithotomy management.

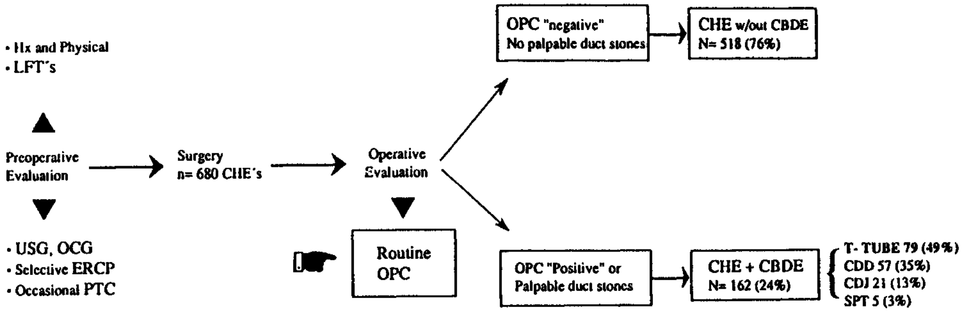

**Figure 1** Diagrammatic outline of the preoperative and intra-operative evaluation methods as well as of the pre and post choledochotomy management utilized by surgeons of Group A (Theory I).

In Group A the routine performance of pre and post exploratory operative cholangiography (OPC) was the mainstay in deciding whether the duct should or should not be explored, in an attempt to minimize the rate of unnecessary CBDE's, and then to make sure that a complete duct clearance had been accomplished. Likewise, still following THEORY I, a temporary T-tube drainage was used in half

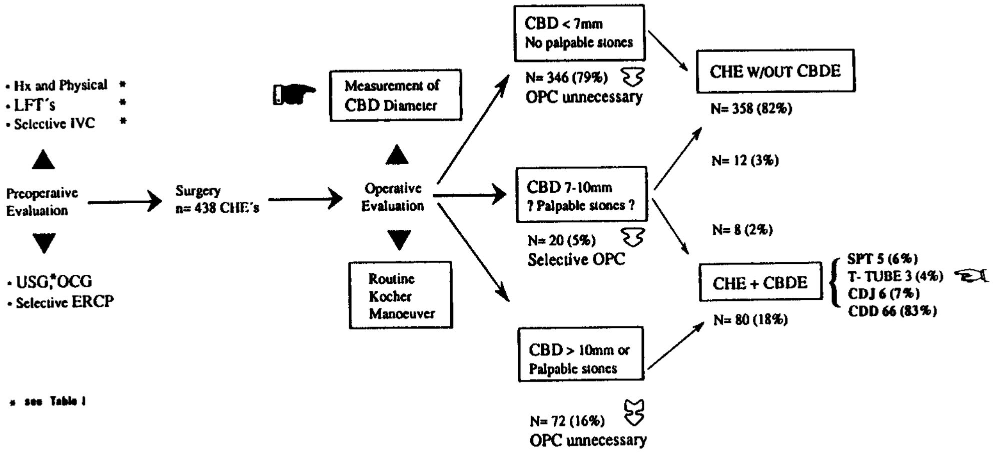

**Figure 2** Diagrammatic outline of the preoperative and intra-operative evaluation methods as well as of the pre and post choledochotomy management in Group B (Theory II).

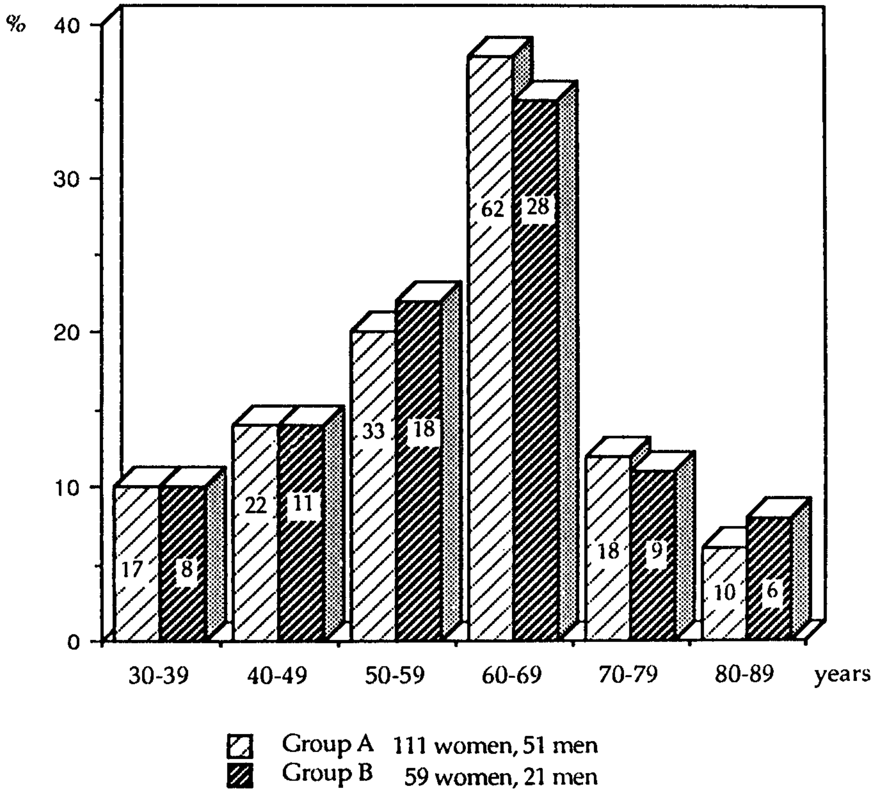

**Figure 3** Age and sex distribution of patients undergoing CBDE in Groups A and B.

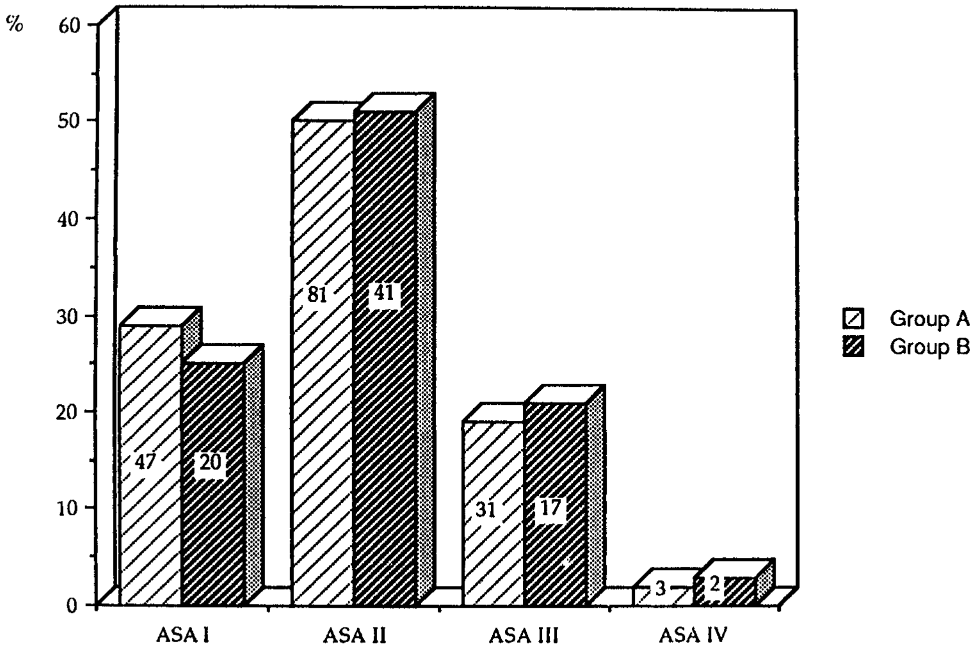

**Figure 4** ASA risk classification of patients undergoing CBDE in Groups A and B.

**Table 1** Criteria establishing the preoperative working diagnosis of papillary (SO) dysfunction (16)

- Upper abdominal pain, related to meals
- Intermittent jaundice
- Biochemical evidence of cholestasis, recent or past
- Stasis of contrast material within a dilated duct (>10 mm) as seen in intra-venous cholangiogram (ICV)
- Dilated common bile duct (>10 mm) as shown by USG.

**Table 2** Indications for common bile duct exploration

| *Absolute* | *Relative* |
|---|---|
| ♦ X-Ray evidence of stones | ♦ Previous episodes of pancreatitis |
| ♦ Palpable calculi | ♦ Multiple GB stones with dilated cystic duct |
| ♦ Present or recent jaundice (Bilirubinemia >7 Mgrms) | ♦ Jaundice (bilirubinemia < = 3 Mgrms) |
| ♦ History of cholangitis | ♦ Thick murky, choledochal bile (gravel) |
| ♦ Papillary "stenosis" | ♦ Thickening of the duct wall |
| ♦ Bilio-enteric fistula | |

of the explored ducts (Figure 1), a definitive decompression having been chosen when doubts persisted about the completeness of the duct clearance. In Group B it was felt that a malfunctioning SO was responsible for the retention of migrated GB calculi or else of "de novo" formation of intra-ductal stones (THEORY II). Therefore, a direct intra-operative measurement, after a wide Kocher manoeuver, of the duct diameter together with extra-ductal palpation were the major factors, complementing the preoperative evaluation, in deciding whether or not to explore the CBD (Figure 2). Common hepatic duct dilatation (> 10 mms), measured with calipers, was taken as indicative of impaired emptying and enhanced likelihood of stones being present. In 20(20/438 or 4.5%) occasions it was felt that OPC was indicated, either because the preoperative work-up (Table 1) raised the suspicion of intra-ductal stones and/or sphincteric dysmotility without any intra-operative confirmation or else because of a dilated duct detected intraoperatively in a patient whose preoperative evaluation had been entirely unhelpful. Stones were revealed by the cholangiogram in 8 such cases. In three of them (3/80 CBDE's or 4%), whose CBD diameter did not exceed 8 mms, a temporary T-tube decompression was undertaken after choledocholithotomy while in the remainder five (5/80 CBDE's or 6%), with a duct width of 9–10 mms, a transduodenal sphincteroplasty was performed. In the remainder 72 CBDE's (72/80 or 90%) (Figure 2) a side-to-side choledochoduodenostomy (CDD)[16] was made in 66 while in 6, because of local factors, a Roux-en-Y choledochojejunostomy (CDJ) was performed.

Pre-discharge T-tube cholangiograms were obtained 8–12 days after surgery. Stones were classified as overlooked/early retained when diagnosed on pre-discharge cholangiograms and as late retained when seen after discharge but within one year post surgery. Calculi causing recurring symptoms after an asymptomatic period of over two years after surgery had elapsed were taken as recurrent.

Only complications causing symptoms and/or delaying the patient's discharge were taken into account in Tables III and IV. A wound infection was defined as one with purulent drainage, regardless of negative bacteriology. Intraperitoneal sepsis was diagnosed by USG examination, CT scanning, relaparotomy or postmortem examination with a clinically suspicious syndrome (intermittent spiking fever, tachycardia, abdominal tenderness, respiratory difficulties, leukocytosis). Acute "ascending" cholangitis was diagnosed by deranged liver biochemistry and USG, with the corresponding clinical syndrome (jaundice, high spiking fever with rigors, sepsis, leukocytosis). The diagnosis of septicaemia was accepted only when a positive blood culture was available. The persistence of bile drainage, through the operative wound or out of a drain site, lasting for more than 5–8 days or its radiographic documentation by dye extravasation, was taken as evidence of biliary fistula. The causes of death were established either at relaparotomy or postmortem examination.

Thorough follow-up data from every patient submitted to CBDE, over one year in all and over 5 years in 110 of Group A and 59 of Group B, were available. The follow-up consisted of a clinical interview, LFT's and USG every 6–12 months[16]. PTC or IVC were obtained selectively. ERCP was routinely obtained, as part of an on-going, long-term, prospective evaluation of the bilio-digestive anastomosis in 25 patients submitted to CDD in Group B, aiming to assess, after 18 months, the stoma width and the presence of food "debris" and/or calculi in the distal "cul-de-sac"[16]. It was also obtained whenever a clinical suspicion of "sumping" and/or cholangitis was raised.

Based on the data collected a classification of long-term results was devised. *EXCELLENT* was freedom from any symptoms and persistently normal LFT's, *GOOD* as occasional or minor gastrointestinal upset or complaints related to wound imperfections with normal LFT's, *FAIR* when significant complaints, abnormal LFT's or endoscopic evidence of pathologic enterogastric reflux, amenable to non-interventional therapy, were documented and *POOR* when "missed"/ residual/recurrent stones, anastomotic complications, jaundice, cholangitis, severely deranged LFT's prompted the need for further invasive, aggressive therapy.

Statistical analyses were obtained, by an independent observer, using the chi-square method (significance level of $X2 >= 3.84$, $P <= 0.05$).

### SHORT TERM RESULTS

There were no significant differences between the Groups with respect to the proportion of ducts undergoing exploration or the "positivity" or "negativity" of such explorations (Figures 5 and 6). Operative morbidity and mortality rates, before and after 30 days from surgery, are shown on Tables 3 and 4. The operative mortality of definitive drainage procedures was higher than T-tube decompression (3.6% versus 1.3%) in Group A, although not reaching statistical significance. The difference is even less significant if the total number of T-tube drainages and definitive decompressions, in both Groups, is considered. Indeed, there was one death occurring after 82 temporary drainages (79 of Group A plus 3 of Group B) for an operative mortality rate of 1.2% and 4 deaths after 160 definitive drainage procedures (83 of Group A plus 77 of Group B) for a mortality rate of 2.5%.

There was a statistically significant difference in morbidity rates, favoring definitive drainage procedures. In four of the temporarily drained ducts, either biliary fistula or acute cholangitis was associated with early retained stones (Table 4). Patients of Group A submitted to CBDE followed by T-tube drainage were not discharged until after an acceptable tube cholangiogram, 8–12 days post operation. However a persistent and significant bile drainage, through the T-tube tract, occurred in 12 of these patients (5 of whom required total parenteral nutrition for periods over two to three weeks).

### LONG TERM RESULTS

In Group A we could demonstrate "missed"/retained/recurrent stones in 10 patients for a 12.7% rate of POOR results (Table 5) in this sub-set of patients. A dilated CBD was recorded as having been present, at surgery, in all of these patients.

Late stenosis of choledocho-jejunal anastomosis in three patients of Group A and an incorrectly placed choledochoduodenal stoma in one patient of Group B account for a 3.8% and 1.3% rate of POOR results, respectively, in patients whose ducts were permanently decompressed. These differences are statistically significant, favoring definitive drainage procedures (Table 5).

Likewise, the rate of EXCELLENT or GOOD results in patients with ducts permanently "fenestrated", just fails to reach statistical significance, compared with those following temporary drainage, in both Groups A and B (Table 5).

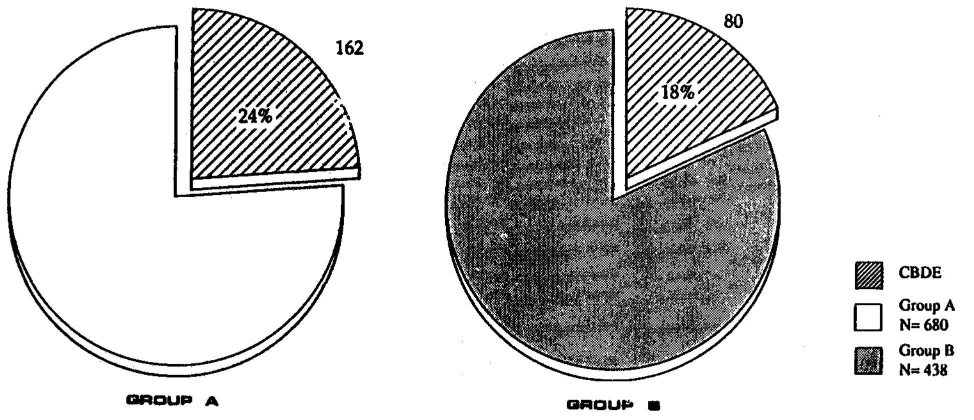

**Figure 5** Percentage of ducts deemed to require exploration (Groups A and B).

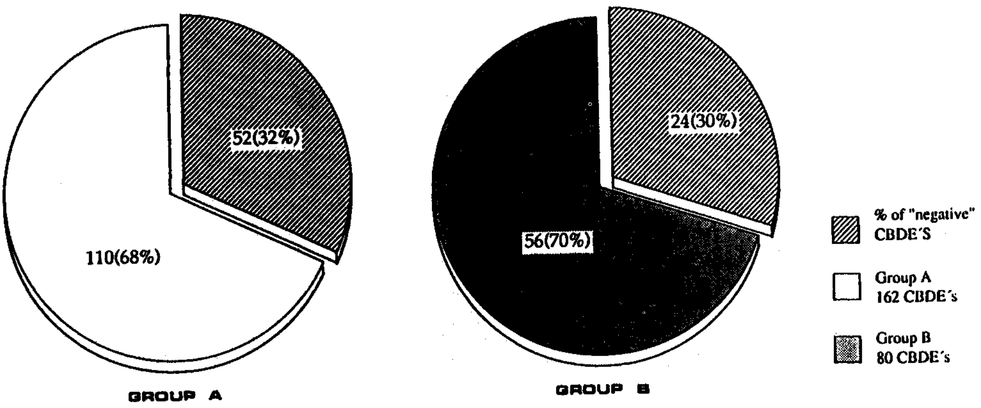

**Figure 6** "Positivity" and "Negativity" of CBDE's in Groups A and B.

### DISCUSSION

It is increasingly evident that GB removal, either by laparotomy or laparoscopically[17-20], remains the most efficient management for a large proportion of patients with symptomatic cholelithiasis. Limited indications exist for the various forms of dissolution therapy and/or ESWL which has a poor cost-effectivness[21] and limited value, in the management of GB stones. Controversy exists over the optimal treatment of the 10 to 20% of patients[10] who have GB and CBD stones particularly when the patient is fit. The controversy revolves around surgical versus endoscopic treatment, and what is the most efficient surgical treatment.

**Table 3** Operative mortality and morbidity rates (Group A and B)

| | *Group A* | | | *Group B* | | |
|---|---|---|---|---|---|---|
| *Operation* | *Nr.* | *Mortality* | *Morbidity* | *Nr.* | *Mortality* | *Morbidity* |
| Cholecystectomy W/out CBDE | 518 | 3 (0.58%) | 41 (7.9%) | 358 | 2 (0.56%) | 21 (5.9%) |
| Cholecystectomy$^+$ CBDE (T-Tube) | 79 | 1 (1.3%)* | 22 (27.8%)$^+$ | 3 | $0^{\S}$ | $0^{0}$ |
| Cholecystectomy$^+$ CBDE (Def. Decompr.) | 83 | 3 (3.6%)** | 10 (12%)$^{++}$ | 77 | 1 (1.3%)$^{\S\S}$ | 6 (7.8)$^{00}$ |
| Total | 680 | 7 (1.02%) | 73 (10.7%) | 438 | 3 (0.68%) | 27 (6.2%) |

Statistical significances: * Versus ** NS ($X^2 = 0.927$). § Versus §§ Non comparable. $^+$ Versus $^{++}$ *Significant* ($X^2 = 6.38$). 0 Versus 00 Non comparable. * Versus § Non comparable. ** Versus §§ Non significant ($X^2 = 0.87$). $^+$ Versus 0 Non comparable. ** Versus §§ Non significant ($X^2 = 0.87$). $^+$ Versus 0 Non comparable. $^+$ Versus 00 *Significant* ($X^2 = 10.9$). $^{++}$ Versus 00 Non significant ($X^2 = 0.80$).

**Table 4** Causes of operative morbidity and mortality and length of Hosp. Stay in patients undergoing CBDE (Group A and B)

| | | *Group A N = 162* | | *Group B N = 80* | |
|---|---|---|---|---|---|
| *Causes of Morbidity and Mortality* | | *T-Tube N = 79* | *Drainage Procedure N = 83* | *T-Tube N = 3* | *Drainage Procedure N = 77* |
| Morbidity | | 22 (27.8%) | 10 (12%) | — | 6 (7.8%) |
| | Biliary Fistula | 5 | 1 | — | 1 |
| | Sepsis, Cholangitis | 2 | — | — | — |
| | Congestive Heart Failure | 1 | 1 | — | 1 |
| "Major" | Deep Vein Thrombosis | 1 | 1 | — | 1 |
| | Pulmonary Embolism | 1 | — | — | — |
| | Atelectasis, Pneumonia | 2 | 1 | — | — |
| | Op. Wound Infection | 8 | 5 | — | 2 |
| "Minor" | Low Urinary Tract Infection | 3 | 1 | — | 1 |
| Mortality | | 1 (1.3%) | 3 (3.6%) | — | 1 (1.3%) |
| ♦ Intra-Peritoneal Sepsis | | 1 | 2 | — | — |
| ♦ Hepato-Renal Syndrome Cholangitis | | — | 1 | — | — |
| ♦ Upper GI Bleeding | | — | — | — | 1 |
| Average Length of Postoperative Hospital Stay (Days) | | 15 (10–25) | 10 (8–21) | — | 7 (5–19) |

It was the purpose of this study to evaluate the short and long term efficiency of two different approaches, when open surgery is the treatment of choice for an individual with gallstones.

The aims of surgery for bile duct stones are: (1) Rid the duct of every single calculus, (2) cause the lowest immediate and long-term morbidity, (3) maintain the integrity of the biliary tree, (4) assure a permanent and efficient duct-duodenal drainage, (5) achieve the above in the same setting incurring the least expenditure and discomfort possible.

**Table 5** Long-term results (*Poor* and *Excellent or good*) in patients undergoing CBDE in Groups A and B, excluding operative mortality

| | *Post CBDE Management* | *Early Ret. Stone* | *Late Ret. Stone* | *Recurrent Stone* | *Anastomotic Complic* | *Poor result* | *Statist Signif* | *Excellent or Good* | *Statist. Signif.* |
|---|---|---|---|---|---|---|---|---|---|
| GROUP A | T-tube (N = 78) | 4 | 3 | 3 | 0 | 10 (12, 7%) | $-X^2 = 4, 3$ | 58 (74%) | $-X^2 = 4.09$ |
| | Definit. Decompr. (N = 80) | — | — | — | 3 | 3 (3, 8%) | $-X^2 = 7.68$ | 68 (85%) | |
| GROUP B | T-tube (N = 30) | — | — | — | — | 0 | −NS | 3 | |
| | Definit. Decompr. (N = 76) | — | — | — | 1 | 1 (1, 3%) | | 66 (87%) | |

Note: Three anastomotic complications (strictures) in Group A observed post Roux-y CDJ and one in Group B post CDD (not a stricture)

The traditional approach to achieve these goals is a supraduodenal choledochotomy and removal of the calculi with the help of operative cholangiography, choledochoscopy or USG. The duct is then closed over a T-tube for 8–12 days decompression. The various modalities of permanent drainage are reserved for a minority of cases with primary impacted ampullary stones, duct dilatation over above 15 mms, the presence of five or more duct stones, low strictures or repeat surgery[10]. However, as reported[22–29], (Table 6), such a policy invariably leads to a significant percentage of "missed"/retained/recurrent stones (5.4% to 20.9%). The results in Group A merely confirm these figures. Toouli[30] has demonstrated by endoscopic manometric measurements, that the predominant pattern of the pressure waves generated within the SO of stone-containing ducts, as opposed to normal controls, is of a retrograde type, encouraging bile stasis in the duct with the resulting favorable "milieu" for primary stone reformation. This lends support to the approach based on THEORY II and pursued in Group B, as previously reported by us and others[12–16]. The superior results, seen in both Groups A and B (Table 6) and in other series[23–25,27–29], following a definitive drainage procedure, as opposed to temporary decompression, also lends support to such an approach. On the other hand the allegedly lower operative morbidity and mortality rates that follow the traditional T-tube approach were not seen in our study. Patients whose ducts were temporarily decompressed had a statistically significantly higher morbidity and in those developing acute cholangitis or biliary fistula, early retained stones could be documented on postoperative cholangiogram and a dilated CBD ($>$ 10 mms) was recorded as having been demonstrated, at surgery, in all of them. It seems to us reasonable to speculate that a correctly performed bilio-digestive "fenestration", by precluding an intra-ductal pressure build-up to a point of causing cholangio-venous reflux[31], would have avoided such complications, even when an intra-ductal stone had been overlooked. Other studies have already emphasized the poorer results ascribable to T-tube management of choledocholithiasis[24–28]. No significant differences could be documented with regard to mortality rates, previous reports comparing, in a prospective randomized fashion, operative morbidity and mortality rates of CDD versus T-tube decompression showed statistically significant differences favoring CDD[24,25]. The Senior Author's total experience, since January 1973, encompasses 102 CDD's as primary surgery for biliary lithiasis — 66 reported here plus 36 in a previous paper[16] — with one operative death.

An argument against the management pursued in Group B, where great reliance is placed on the intraoperative measurement of the duct diameter as the most significant indicator for choledochotomy, would be a prohibitively high proportion of "negative" CBDE's and of "missed" stones. However, as Figures 5 and 6 point out, these rates were similar in both Groups and within the range commonly reported as acceptable[32].

It is also argued that a side-to-side CDD or CDJ would create a distal "cul-de-sac" where food "debris" and/or retained/reformed stones would accumulate, known as the "sump" syndrome. However, as previously reported[16], whenever a wide enough (at least 2.5 cms) choledocho-enteric anastomosis is properly constructed no such syndrome occurs. Indeed, in only 4 patients, out of 156 (2.6%) submitted to such drainage procedures and assessed by ERCP, more than 18 months post surgery, could we document anastomotic stenosis, with food "debris", in three and a poorly located stoma (not stenotic), high above the cystic duct confluence hindering free flow of bile in one. These are the patients reported as

**Table 6** Rates of early retained, late retained and recurrent choledochal stones after standard surgical CBDE followed by either temporary T-Tube drainage or definitive decompression (drainage procedure)

| | | *T-Tube* | | | | *Drainage procedure* | | | | | *Stat. Signif.* |
|---|---|---|---|---|---|---|---|---|---|---|---|
| | *Nr. CBDE* | *Ear Ly* | *Late* | *Recurr* | *Total* | *Nr.* | *Early* | *Late* | *Recurr* | *Total* | |
| Way (22) 1972 | 200 | 3 | 5 | 6 | 14 (7%) | 0 | — | — | — | — | — |
| Rattner and Warshow (23) 1981 | 421 | 7 | 12 | 4 | 23 (5.4%) | 78 | 0 | 0 | 0 | 0 | Sign ($X^2=4.46$) |
| Lygidakis (24) 1982) | 16 | — | — | 2 | 2 (12.5%) | 26 | 0 | 0 | 0 | 0 | NS ($X^2=3.4$) |
| Lygidakis (25) 1983 | 43 | — | — | 9 | 9 (20.9%) | 45 | 0 | 0 | 0 | 0 | Sig ($X^2=10.49$) |
| Rogers (26) 1985) | 100 | 9 | — | — | 9 (9%) | 0 | — | — | — | — | — |
| Crumplin (27) 1985 | 56 | — | 7 | — | 7 (12.5%) | 100 | 0 | 1 | 0 | 1 (0.9%) | Sign ($X^2=9.7$) |
| Sheridan (28) 1987 | 209 | 20 | 7 | 10 | 37 (17.7%) | 48 | 0 | 0 | 0 | 0 | Sign ($X^2=9.9$) |
| Neoptolemos (29) 1987 | 191 | 13 | 13 | — | 26 (13.6%) | 95 | 0 | 0 | 0 | 0 | Sig ($X^2=14.22$) |
| Total | 1236 | 52 | 44 | 31 | 127 (10.2%) | 392 | 0 | 1 | 0 | 1 (0.25%) | |
| Present Series Group A | 78 | 4 | 3 | 3 | 10 (12.8%) | 80 | 0 | 0 | 0 | 0 | Sign ($X^2=4.3$) |
| Present Series Group B | 3 | 0 | 0 | 0 | 0 | 76 | 0 | 0 | 0 | 0 | Non Comparable |

Note: Operative mortality was not taken into account. Routine OPC or choledochoscopy or both were utilized by the Authors of the reports herein quoted.

having had POOR long term results after drainage procedures which compare favorably with the rate of POOR results (10 out of 81 or 12.3%) following T-tube, decompression, Table 5.

It might seem inappropriate to lump together early retained stones with late recurring ones, as observed in patients of Group A submitted to temporary decompression and, then, compare their rate of POOR results with that observed among patients with ducts definitively drained, because no investigation was carried out aiming to assess the occurrence of early retained/"missed" stones following the latter group. We would point out that it was the purpose of this study to evaluate the total number of patients requiring further aggressive therapy — surgical, endoscopic or simple stone extraction via the T-tube tract, regardless of when it occurred in the postop course. Secondly we would stress out that the correct performance of a drainage procedure precludes the need to look for "missed"/early retained stones because the construction of a wide enough stoma will prevent any possible "missed" or irretrievable calculus causing morbidity since it will pass easily across the anastomosis or else will avoid any dangerous build-up of intra-biliary pressure. This is not the case with temporary, T-tube, decompressions after which, with or without the additional help of OPC or choledochoscopy, the occurrence of early retained/"missed" stones keeps being reported (22–29, Table 6). Over the past decade, a number of reports have shown the poor efficiency of routine OPC[33–36] while some others[33,37,38] have come to emphasize that a dilated CBD represents the most significant indicator of the presence of stones.

Although the availability of sophisticated endoscopic techniques enables us to solve many of these early and long-term complications, they should not prevent a complete, optimal, operation. It is important to remember that each non-operative procedure has its own early and long-term morbidity and mortality risks. The goal is to rid the patient of stones with the fewest procedures and the lowest risk of morbidity and death.

The role to be played by the newer technologies, namely laparoscopic CHE accompanied by EST must be found in large scale, randomized, controlled studies comparing the laparoendoscopic approach to that pursued by Group B, which is the surgical management that has been shown, in this study, to be the most efficient in accomplishing the goals previously mentioned.

### ABBREVIATIONS

| | |
|---|---|
| EST | Endoscopic Sphincterotomy |
| ESWL | Extra-Corporeal Shock Wave Lithotripsy |
| CBD | Common Bile Duct |
| GB | Gallbladder |
| CBDE | Common Bile Duct Exploration |
| CHE | Cholecystectomy |
| LFT | Liver Function Tests |
| IVC | Intra-Venous Cholangiography |
| USG | Ultrasonography |
| SO | Sphincter of Oddi |
| OPC | Operative Cholangiography |

| | |
|---|---|
| CDJ | Choledochojejunostomy |
| CDD | Choledochoduodenostomy |
| ERCP | Endoscopic Retrograde Cholangio Pancreatography |
| PTC | Percutaneous Transhepatic Cholangiography |

### *References*

1. Classen, M., Demling, L. (1974) Endoscopische sphinkterotomie der papilla vateri Disch. *Med. Wochenschr.*, **99**, 496–497
2. Kawai, K., Akasaka, Y., Murakami, M. (1974) Endoscopic sphincterotomy of the ampulla of vater. *Gastrointest. Endosc.*, **20**, 148–151
3. Neoptolermos, J.P., Shaw, D.E. and Carr-Locke, D.L. (1989) A multivariate analysis of preoperative risk in patients with common bile duct stones — Implications for treatment. *Ann. Surg.*, **209**, 157–161
4. Davidson, B.R., Neoptolemos, J.P. and Carr-Locke, D.L. (1988) Endoscopic Sphincterotomy for common bile duct calculi in patients with gallbladder "in situ" considered unfit for surgery. *Gut*, **29**, 114–120
5. Tanaka, M., Ikeda, S., Yoshimoto, H. and Matsumoto, S. (1987) The long-term fate of the gallbladder after endoscopic sphincterotomy. Complete follow-up study of 122 patients. *Am. J. Surg.*, **154**, 505–509
6. Olaison, G., Kald, B., Karlqvuist, P.A., Lindstrom, E. and Anderberg, B. (1987) Endoscopic removal of common bile duct stones without subsequent cholecystectomy. *Acta Chir. Scand.*, **153**, 541–543
7. Worthley, C.S. and Toouli, J. (1988) Gallbladder non-filling: an indication for cholecystectomy after endoscopic sphincterotomy. *Br. J. Surg.*, **75**, 796–798
8. Miller, B.M., Kozarek, R.A., Ryan, Jr. J.A., Ball, T.J., and Traverso, L.W. (1988) Surgical versus endoscopic management of common bile duct stones. *Ann. Surg.*, **207**, 135–141
9. Graham, S.M., Krige, J.E.J., and Bornman, P.C. (1989) Biliary surgery: stones and strictures. *Current Opinion Gastroenterology*, **5**, 642–643
10. Johnson, A.G., and Hosking, S.W. (1987) Appraisal of the management of bile duct stones. *Br. J. Surg.*, **74**, 555–560
11. Vennes, J.A. (1983) Management of calculi in the common duct. *Seminars in liver disease*, **3**(2), 162–171
12. Madden, J.L., Chun, J.Y., Kandalaft, S., and Parek, H.M. (1970) Choledochoduodenostomy: an "unjustly" maligned operation. *Am. J. Surg.*, **119**, 45–54
13. Madden, J.L. (1973) Common duct stones: their origin and surgical management. *Surg. Clin. North Am.*, **53**, 1095–1113
14. Taylor, T.V. and Armstrong, C.P. (1987) Migration of gall stones. *Br. Med. Journal*, **294**, 1320–1322
15. Almeida, A.M., Gracias, C.W. and Santos, N.M. (1982) Definitive decompression of the biliary tree-perferred approach to management of choledocholithiasis. *Am. J. Gastroent.*, **77**, 941–946
16. Almeida, A.M., Cruz, A.G. and Aldeia, F.J. (1984) Side-to-side choledochoduodenostomy in the management of choledocholithiasis and associated pathology — Facts and fiction. *Am. J. Surg.*, **147**, 253–258
17. Gadacz, T. and Talamini, M. (1991) Traditional versus laparoscopic cholecystectomy. *Am. J. Surg.*, **161**, 336–338
18. Spaw, A.T., Reddick, E.J. and Olsen, D.O. (1991) Laparoscopic laser cholecystectomy: analysis of 500 procedures. *Surg. Laparosc. and Endosc.*, **1**, 2–7
19. Olsen, D.O. (1991) Laparoscopic cholelystectomy, *Am. J. Surg.*, **161**, 339–334
20. Quattlebaum, Jr. J.K. and Flanders, H.D. (1991) Laparoscopic of common bile duct stones. *Surg. Laparosc. and Endosc.*; **1**, 26–32
21. Pitt, H.A. (1972) XIV International course of surgery, Madrid, Spain (E.M. Gonzalez), May, 20–25, 1991 (Anaitting Publication) 22 — Way L.W., Admirand, W.H., Dunphy, J.E., Management of choledocholithiasis. *Ann. Surg.*, **176**, 347–357
22. Way, L.W., Admirand, W.H. and Dunphy, J.E. (1972) Management of choledocholithiasis *Ann. Surg.*, **176**, 347–357
23. Rattner, D.W., Warshaw, A.L. (1981) Impact of Choledochoscopy on the management of choledocholithiasis. *Ann. Surg.*, **194**, 76–79

24. Lygidakis, N.J. (1982) Acute suppurative cholangitis: comparison of internal and external biliary drainage. *Am. J. Surg.*, **143**, 304–306
25. Lygidakis, N.J. . (1983) Surgical approach to recurrent choledocholithiasis-choledochoduodenostomy versus T-tube drainage after choledochotomy. *Am. J. Surg.*, **145**, 636–639
26. Rogers, A.L., Farha, G.F., Beamer, R.L. and Chang, F.C. (1985) Incidence and associated mortality of retained common bile duct stones. *Am. J. Surg.*, **150**, 690–693
27. Crumplin, M.K.H., Jenkinson, L.R., Kassab, J.Y., Whitaker, C.M. and al Boutiahi, F.H. (1985) Management of gallstones in a district general hospital. *Br. J. Surg.*, **72**, 428–432
28. Sheridan, W.G., Williams, H.O.L. and Lewis, M.H. (1987) Morbidity and mortality of common bile duct exploration. *Br. J. Surg.*, **74**, 1095–1099
29. Neoptolemos, J.P., Davidson, B.R., Shaw, D.E., Lloyd, D., Carr-Locke, D.L. and Fossard, D.P. (1987) Study of common bile duct exploration and endoscopic sphincterotomy in a consecutive series of 438 patients. *Br. J. Surg.*, **74**, 916–921
30. Toouli, J., Geenen, J.E. and Hogan, W.J. (1982) Sphincter of oddi motor activity: a comparison between patients with common bile duct stones and controls. *Gastroenterology*, **82**, 111–117
31. Huang, T., Bass, T.A. and Williams, R.D. (1969) The significance of biliary pressure in cholangitis. *Arch. Surg.*, **98**, 629–634
32. Den Besten, L. and Berci, G. (1986) The current status of biliary tract surgery: an international study of 1072 consecutive patients. *World J. Surg.*, **10**, 116
33. Taylor, T.V., Torrance, B., Rimmer, S., Hillier, V., Lucas, S.B. (1983) Operative cholangiography: is there a statistical alternative? *Am. J. Surg.*, **145**, 640–643
34. Gerber, A., APT, M.K. (1982) The case against routine operative cholangiography, *Am. J. Surg.*, **143**, 734–736
35. Gregg, R.O. (1988) The case for selective cholangiography. *Am. J. Surg.*, **155**, 540–544
36. Pernthaler, H., Sandblicher, P., Schmid, T.H. and Margreiter, R. Operative cholangiography in elective cholecystectomy. *Br. J. Surg.*, **77**, 339–400
37. Reiss, R., Deutsch, A.A., Nudelman, I. and Kott, I. (1984) Statistical value of various clinical parameters in predicting the presence of choledochal stones. *Surg. Gyn. Obstet.*, **159**, 273–276
38. Taylor, T.V., Armstrong, C.P., Rimmer, S., Lucas, S.B., Jeacock, J. and Gunn, A.A. (1988) Prediction of choledocholithiasis using a pocket microcomputer. *Br. J. Surg.*, **75**, 138–140

*(Accepted by S. Bengmark 28 January 1992)*

